# Outside-in repair technique is effective in traumatic tears of the meniscus in active adults: a systematic review

**DOI:** 10.1007/s00167-023-07475-z

**Published:** 2023-06-14

**Authors:** Filippo Migliorini, Marco Pilone, Andreas Bell, Michael Celik, Christian Konrads, Nicola Maffulli

**Affiliations:** 1 Department of Orthopaedic, Trauma, and Reconstructive Surgery, RWTH University Hospital, Pauwelsstraße 30, 52074 Aachen, Germany; 2 Department of Orthopaedics and Trauma Surgery, Academic Hospital of Bolzano (SABES-ASDAA), 39100 Bolzano, Italy; 3 Residency Program in Orthopedics and Traumatology, University of Milan, Milan, Italy; 4 Department of Medicine, Surgery and Dentistry, University of Salerno, 84081 Baronissi, SA Italy; 5 Department of Orthopaedic and Trauma Surgery, Eifelklinik St. Brigida, 52152 Simmerath, Germany; 6 Department of Orthopaedics and Traumatology, Helios Hanseatic Hospital Stralsund, Stralsund, Germany; 7 Medical Faculty, University of Tübingen, 72076 Tübingen, Germany; 8 School of Pharmacy and Bioengineering, Faculty of Medicine, Keele University, ST4 7QB Stoke On Trent, England; 9 Queen Mary University of London, Barts and the London School of Medicine and Dentistry, Centre for Sports and Exercise Medicine, Mile End Hospital, E1 4DG London, England

**Keywords:** Meniscus, Meniscal injuries, Meniscal repair, Outside-in

## Abstract

**Purpose:**

Meniscal injuries are common. Outside-in meniscal repair is one of the techniques advocated for the management of traumatic meniscal tears. This systematic review investigated the outcomes of the outside-in repair technique for the management of traumatic tears of the menisci. The outcomes of interest were to investigate whether PROMs improved and to evaluate the rate of complications.

**Methods:**

Following the 2020 PRISMA statement, in May 2023, PubMed, Web of Science, Google Scholar, and Embase were accessed with no time constraints. All the clinical investigations which reported data on meniscal repair using the outside-in technique were considered for inclusion. Only studies which reported data on acute traumatic meniscal tears in adults were considered. Only studies which reported a minimum of 24 months of follow-up were eligible.

**Results:**

Data from 458 patients were extracted. 34% (155 of 458) were women. 65% (297 of 458) of tears involved the medial meniscus. The mean operative time was 52.9 ± 13.6 min. Patients returned to their normal activities at 4.8 ± 0.8 months. At a mean of 67-month follow-up, all PROMs of interest improved: Tegner scale (*P* = 0.003), Lysholm score (*P* < 0.0001), International Knee Documentation Committee (*P* < 0.0001). 5.9% (27 of 458) of repairs were considered failures. Four of 186 (2.2%) patients experienced a re-injury, and 5 of 458 (1.1%) patients required re-operation.

**Conclusion:**

Meniscal repair using the outside-in technique can be effectively performed to improve the quality of life and the activity level of patients with acute meniscal tears.

**Level of evidence:**

Level IV.

## Introduction

The menisci are wedge-shaped fibrocartilages which ensure smooth articulation and redistribution of load within the tibiofemoral joint. Structurally, menisci consist predominantly of type I collagen in addition to proteoglycans and elastin [18, 65]. Shock absorption during gait and increasing joint stability are other important functions of the menisci [45]. The lateral and medial menisci differ in shape, percentage of tibial plateau coverage, and load transfer on the medial and lateral compartments during distinct knee movements [30, 34]. The approximate estimated incidence of symptomatic meniscal tears is 60 per 100,000 people [2, 7]. Male adults older than 40 years are more at risk to develop degenerative meniscal tears [19, 35]. Acute meniscal injuries are more prevalent in younger and active patients [6, 35, 65]. Management of meniscal tears depends on patient characteristics and the aetiology, morphology, and location of the tear [4, 7]. In patients with symptomatic meniscal tears refractory to conservative management or in those with mechanical symptoms, arthroscopy may be recommended [33, 43, 63]. When possible, meniscal repair is advocated over meniscectomy [32, 66]. Compared to meniscal repair, meniscectomy is associated with worse outcomes, faster osteoarthritis progression, and lower midterm cost-effectiveness [14, 15, 44, 52].

All-inside, inside-out, and outside-in meniscal repair are the most common techniques of meniscal repair [58, 61]. The outside-in technique was first described by Warren et al. to decrease the risk of peroneal nerve injury [64]. The most common indication is an anterior horn tear, given the difficulty of reaching this area using the all-inside technique [37, 53]. The lesion must be in the red-red or red-white zone, although successful meniscal repairs have been described in the white–white zone using fibrin clot augmentation [13, 36, 60]. The surgical technique entails passing two needles, from outside inward, through the capsule and the meniscal tear [29, 51]. One needle carries a loop of thread or metal, and the other the suture [28, 50, 68]. The most common complications are stiffness, failure of meniscal healing, and neurovascular damage [21, 39]. To the best of our knowledge, an updated systematic review on the efficacy and safety of outside-in meniscal repair is missing. Therefore, this systematic review investigated the outcomes of the outside-in repair technique for traumatic tears of the menisci. The outcomes of interest were to investigate whether the outside-in repair is associated with an improvement in PROMs and to evaluate the rate of complications.

## Material and methods

### Eligibility criteria

All the clinical investigations which reported data on meniscal repair using the outside-in technique were considered for inclusion. Studies which reported data on other meniscal repair methods (inside-out, all-inside) or arthroscopic meniscectomy (partial or total) were not suitable. Given the author’s language capabilities, articles in English, German, Italian, French, and Spanish were eligible. Only studies with levels I to IV of evidence, according to the Oxford Centre of Evidence-Based Medicine [26], were considered. Commentaries, abstracts, revisions, opinions, editorials, and letters were not eligible. Biomechanical studies on cadavers or animals were not eligible, nor were in vitro studies. Only studies which reported data on traumatic meniscal tears in adults were considered. Studies which reported data on degenerative tears or on adults older than 45 years were not considered. Only studies which reported a minimum of 24 months of follow-up were eligible. Missing quantitative data on the outcomes of interest warranted the exclusion from the present investigation.

### Search strategy

This study followed the Preferred Reporting Items for Systematic Reviews and Meta-Analyses: the 2020 PRISMA statement [47]. The PICOT algorithm was preliminarily established:

- P (Problem): traumatic meniscal tears;
- I (Intervention): meniscal repair;
- C (Comparison): outside-in technique;
- O (Outcomes): PROMs, rate of re-injury, failure, and revision surgery.
- T (Timing): minimum 24 months follow-up.

In May 2023, PubMed, Web of Science, Google Scholar, and Embase were accessed with no time constraints. These keywords were given in the search bar of each database using the Boolean operator AND/OR as follows: (meniscus OR meniscal OR menisci) AND (injury OR tear OR rupture OR torn OR laceration) AND outside-in AND (PROMs OR outcome OR surgery OR Tegner OR Lysholm OR IKDC OR pain OR symptoms OR complications OR failure OR reoperation). No additional filters were for the search.

### Selection and data collection

Two authors (MP and MC) independently performed data selection. All the resulting titles were screened by hand. If the titles matched the topic, the abstract was accessed. If the abstract matched the topic, the full text of the article was accessed. If the full text was not accessible, the article was excluded. The bibliographies were also screened by hand to identify further studies. All the resulting articles were assessed, and their eligibility was discussed. In case of disagreements, a third author took the final decision (NM).

### Data items

Two authors (MP and MC) separately performed data extraction. The study generalities and the patient demographic at baseline (author, year of publication, journal, mean length of the follow-up, number of patients, mean age, mean BMI) were collected. Data concerning the following PROMs were collected at baseline and at the last follow-up: Tegner Activity Scale [10], Lysholm Knee Scoring Scale [40], and International Knee Documentation Committee (IKDC) [25]. The minimum clinically important difference (MCID) for the Lysholm score was 10/100, 15/100 for the IKDC, and 0.5/10 for the Tegner score [3, 27, 46]. The rate of complications (re-tear, re-operations, failure) was also collected. Failures were defined as the presence of symptomatic re-tears which impair the quality of life and sport participation and required additional surgery.

### Assessment of the risk of bias

To assess the methodological quality, the Coleman Methodology Score (CMS) was used. The CMS is divided into parts A and B. The first part evaluated the study size, mean follow-up, surgical approach, type of study, description of the diagnosis, surgical technique, and post-operative rehabilitation. The second part evaluated the outcome criteria, the procedure for assessing outcomes, and the description of the subject selection process. The CMS resulted in a value between 0 (poor quality) and 100 (excellent quality). Values of CMS > 60/100 are considered satisfactory.

### Statistical analysis

The statistical analyses were performed by the main author (FM) following the recommendations of the Cochrane Handbook for Systematic Reviews of Interventions [24]. For descriptive statistics, mean and standard deviation were used. To evaluate the improvement from baseline to the last follow-up, the SPSS software was used. The mean difference (MD) was calculated, with 95% confidence interval (CI). The paired t-test was performed with values of P < 0.05 considered statistically significant.

## Results

### Study selection

**Fig. 1:**
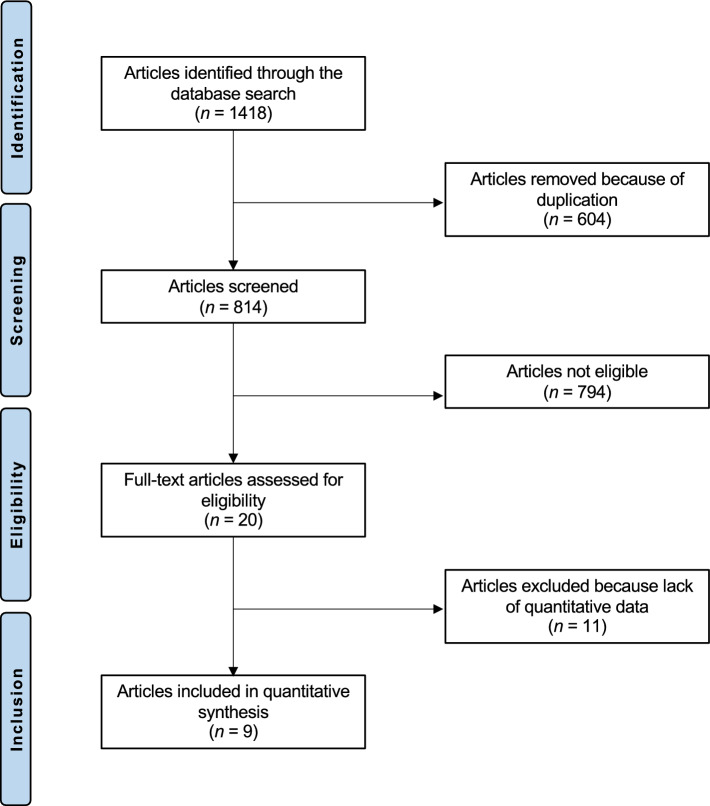
PRISMA flow chart of the literature search

The literature search resulted in 1,418 studies. Of them, 604 were excluded as they were duplicates. A further 794 studies were excluded as they did not match the eligibility criteria: not matching the topic (*N* = 278), not reporting data on meniscal repair using the outside-in technique (*N* = 179), inappropriate study design/study type (*N* = 311), reporting data on degenerative tears or reporting data on adults older than 45 years (*N* = 11), follow-up shorter than 24 months (*N* = 9), language limitations (*N* = 6). A further 11 studies were excluded as they did not report quantitative data on the outcomes of interest. Finally, nine clinical investigations were included. The results of the literature search are shown in Fig. 1.

### Risk of bias assessment

The study size and the length of the follow-up were appropriate in most articles. All authors investigated only the outside-in technique. 66% (6 of 9) of studies were retrospective, and 33% (3 of 9) were prospective. Moreover, no study was randomised, increasing the risk of selection bias. The description of diagnosis and surgical technique was adequate in most studies, whereas information on post-operative rehabilitation was barely reported. The outcome criteria and procedures for assessing outcomes were reliable in most studies. The description of the subject selection process was often adequately described. The CMS resulted in 66 ± 8 points, attesting a good quality of the methodology. The CMS related to each study is reported in Table 1.

**Table 1:** Generalities and patient baseline of the included studies (CMS: Coleman Methodology Score)

Author, year	Journal	Design	CMS	Follow-up (*months*)	Patients (*n*)	Medial side (*n*)	Mean age	Women (*n*)
Biedert et al. 2000 [8]	Knee Surg Sports Traumatol Arthrosc,	Prospective	73	27	40	41	30.4	19
Brucker et al. 2011 [11]	Knee Surg Sports Traumatol Arthrosc,	Prospective	75	247	45	15	20.6	19
Domzalski et al. 2021 [13]	J Orthop Surg	Retrospective	61	37	92	77	31.5	56
Lee et al. 2019 [38]	J Orthop Surg (Hong Kong)	Retrospective	52	24	70	47	27.0	0
Majewsk et al. 2006 [41]	Am J Sport	Retrospective	54	120	88	50	29.8	34
Marinescu et al. 2003 [42]	Knee Surg Sports Traumatol Arthrosc	Retrospective	64	60	68	51	27.6	11
Pogorelić et al. 2020 [50]	Acta Clin Croat	Retrospective	58	40	18	13	17.0	7
Raoulis et al. 2021 [51]	Cureus	Retrospective	66	24	8	3	25.3	1
Zhuo et al. 2020 [68]	BMC Muskuloskel, Disorders	Prospective	61	27	29	0	25.4	8

### Study characteristics and results of individual studies

Data from 458 patients were extracted. 34% (155 of 458) were women. The mean length of follow-up was 67.3 ± 74 months. 65% (297 of 458) of the meniscal tears were medial. The mean operative time was 52.9 ± 13.6 min. Patients returned to their normal activities at 4.8 ± 0.8 months from the index procedure. The generalities and demographics of the included studies are shown in Table 1.

### Synthesis of results

At last follow-up, all PROMs of interest were statistically improved (Table 2): Tegner scale (MD 0.7; *P* = 0.003), Lysholm score (MD 21.4; *P* < 0.0001), IKDC (MD 28.9; *P* < 0.0001).

**Table 2:** Main results of PROMs

PROM	At baseline	At last FU	MD	SE	95% CI	*P*
Tegner activity scale	4.7 ± 2.2	5.4 ± 0.7	0.7	0.23	0.24 to 1.15	0.003
Lysholm knee scoring System	71.7 ± 5.1	93.1 ± 3.8	21.4	0.63	20.14 to 22.65	< 0.0001
IKDC	58.9 ± 18.4	87.8 ± 4.5	28.9	1.89	25.16 to 32.63	< 0.0001

*FU* follow-up, *SE* standard error, *CI* confidence of interval, *PROM* patient-reported outcome measure, *IKDC* International Knee Documentation Committee

### Complications

5.9% (27 of 458) or repair resulted in failures. The re-injury rate was 2.2% (4 of 186), and 1.1% (5 of 458) of patients required a re-operation.

## Discussion

According to the main findings of the present study, meniscal repair using the outside-in technique achieves a statistically significant improvement in the Tegner Activity Scale, Lysholm Knee Scoring Scale, and IKDC. The improvement in PROMs overcome their MCID in all comparisons [3, 27, 46], but in 27 of 458 patients (5.9%) failures occurred. Four of 186 (2.2%) patients experienced a re-injury, and 5 of 458 (1.1%) patients required re-operation. However, among the included studies, only a few articles reported information on complications, and the real safety of the outside-in meniscal repair remains not fully clarified.

Biedert et al. [8] studied 40 patients, divided into four groups, based on the treatment received. Conservative management, arthroscopic suture repair, partial meniscectomy and partial meniscectomy combined with fibrin clot. A statistically significant improvement in functional scores was found in the suture repair group compared with the conservatively managed group. Patients who had undergone partial meniscectomy had better clinical outcomes than the meniscal suture group, and no patient suffered any post-operative complications. These outcomes can be related to the length of the follow-up [8]. The short-term outcomes are best in partial meniscectomy, but partial meniscectomy is associated with osteoarthritis progression [1, 16, 52, 55]. Lee et al. [38] studied meniscal sutures and partial meniscectomy, comparing the 18 months of follow-up and the follow-up after 18 months. At early follow-up, partial meniscectomy showed better clinical outcomes than meniscal sutures. At late follow-up, the IKCD and Tegner score were significantly better in the meniscal suture group than in the partial meniscectomy group. The scores of partial meniscectomy tended to decline over time, while the scores of meniscal suture remained stable. A partial meniscectomy removes the origin of the pain immediately after the surgery, while a meniscal suture requires time for healing [38]. Better clinical outcome was evident in patients undergoing simultaneous meniscal repair and ACL reconstruction than in patients having an isolated meniscal repair. The release of cytokines and growth factors after the drilling of bone tunnels could improve meniscal healing [22, 56]. Pogorelic et al. [50] conducted a study on adolescents and analysed the result of outside-in suturing and all-inside dart fixation. Excellent results were obtained in both groups, and clinical outcomes were comparable. Dart fixation is used preferably in posterior horn lesions, but darts can cause cartilage injury, while outside-in suturing is used preferably in anterior horn lesions [51, 57]. Majewski et al. [41] analysed the long-term effects of meniscal repair in 88 patients with a mean follow-up of 10 years (5–17 years). In 24% of patients, a traumatic or degenerative meniscal re-tear occurred. A statistically significant difference was found in the progression of osteoarthritis between the injured and the non-injured knee.

The rate of knee osteoarthritis after meniscal repair is higher compared to the general population [48]. However, in a study with over 20 years of follow-up, the longest follow-up to date, there was no statistically significant difference in osteoarthritis progression between the operated knee and the contralateral knee [11]. No signs of knee malalignment were found in the operated knees [11]. Two studies evaluated meniscal healing using post-operative MRI [51, 68]. Cereus et al. [51] showed healing in 7 of 8 patients, according to clinical tests and imaging, after 24 months of follow-up. In Zhou et al. study [68], post-operative MRI showed complete healing in 28 of 29 patients. A second-look arthroscopy was performed on 22 patients after 13 months. A total of 19 patients showed complete healing and 3 patients partial healing. No failure of healing was found. Domzalsky et al. [13] analysed the influence of smoking on meniscal healing. A prolonged time of return to daily and sport activities and worst functional scores were found among smokers [9]. Blood supply during meniscal healing is compromised in smokers [5, 20, 59].

The present systematic review has several limitations. The retrospective nature of most studies, along with the limited sample size and length of follow-up, represent important limitations. Between studies heterogeneities are evident. Three studies excluded patients with a concomitant ACL injury [13, 41, 51]. As stated above, ACL reconstruction favourable influences meniscal healing [38]. The location of meniscal tears was not homogeneous among the studies. Two studies analysed only anterior horn tears [38, 51]. One study analysed only lateral meniscus posterior root tears [68]. The outside-in technique is the most appropriate for anterior horn tears because it allows a direct approach to the lesion and a stable fixation construct [53]. One study included only longitudinal meniscal tears [41]. Characterisation of a meniscal tear is important because vertical and longitudinal tears are most suitable for outside-in suture [62]. In one study, an arthrotomy was used [11]. The longest-term follow-up of this study permits a comprehensive view of outside-in long-term results. Zhou et al. [68] utilised a side-to-side surgical technique. This technique allows an anatomic repair and does not change the meniscus physiological properties after a posterior horn lesion [31]. The literature shows little agreement on guidelines on rehabilitation after meniscal repair [12, 23, 54, 67]. Weight-bearing was not allowed in four of the included studies for the first 2–6 weeks [13, 38, 51, 68]. In two studies, partial weight bearing was allowed for the first 6 weeks [8, 41]. In two studies, full weight bearing was allowed immediately after surgery [11, 42]. Limitation in knee flexion was present in all the rehabilitation protocols, for 2–6 weeks, involving a gradual return to total knee flexion. Pogorelic et al. [50] did not specify their rehabilitation protocol. A recent systematic review analysed the rehabilitation protocol in 88 studies after meniscal repair [17]. In two-thirds of the included studies, partial weight bearing was allowed within the first week. In 23.4% of the studies, full weight bearing was allowed at 6 weeks after surgery. In one-third of the studies, full flexion was allowed at 6 weeks after surgery. Only three studies presented over 5 years of follow-up [11, 41, 42]. The prevalence of meniscal re-rupture can be influenced by the length of follow-up [49].

Concluding, the present systematic review indicates that meniscal repair using the outside-in technique can be effectively performed to improve the quality of life and the activity level of patients with acute meniscal tears. However, additional investigations are required to properly establish the safety profile of such procedure.

## Conclusion

Meniscal repair using the outside-in technique was associated with an improvement in the Tegner Activity Scale, Lysholm Knee Scoring Scale, and IKDC. 5.9% were considered failures. Four of 186 (2.2%) patients experienced a re-injury, and 5 of 458 (1.1%) patients required re-operation.

## Data Availability

The data underlying this article are available within the article.

## Abbreviations

FU: Follow-up
SE: Standard error
CI: Confidence of interval
PROM: Patient-reported outcome measure
IKDC: International knee documentation committee
MD: Mean difference
CMS: Coleman methodology score
MCID: Minimum clinically important difference
